# A new copper-supported zinc ferrite as a heterogeneous magnetic nanocatalyst for the synthesis of bis(pyrazolyl)methanes and oxidation of sulfides

**DOI:** 10.1038/s41598-022-25170-w

**Published:** 2022-12-01

**Authors:** Arash Ghorbani-Choghamarani, Hamid Aghavandi, Seyed Mahdi Talebi

**Affiliations:** Department of Organic Chemistry, Faculty of Chemistry, Bu-Ali Sina University, Hamedan, 6517838683 Iran

**Keywords:** Heterogeneous catalysis, Magnetic materials

## Abstract

In this paper, we report the synthesis of ZnFe_2_O_4_@SiO_2_@APTES@DHBS-Cu as a novel magnetic nanocatalyst, in a mild and green environment. The structure of the described magnetic compound was characterized by different physicochemical techniques including XRD, EDS, AAS, SEM, FT-IR, X-ray elemental mapping, TGA, and VSM analyses. The prepared magnetic nanoparticles exhibit excellent catalytic activity in synthesizing bis (pyrazolyl)methanes and oxidation of sulfide derivatives under green conditions. The heterogeneous nature of the catalyst was confirmed via the hot filtration experiment. Further, ZnFe_2_O_4_@SiO_2_@APTES@DHBS-Cu showed high efficiency and reusability that could be reused for at least five consecutive runs.

## Introduction

In the past decade, some specific efforts have been made to design effective magnetic nanoparticles instead of immobilized precious metals on mesoporous silica materials^1,2^. In catalytic studies, the recovery and reusability of the catalyst due to its adaptability to environmental concerns are important features of the catalytic process that has received much attention in recent years^3–7^. The recovery and reusability of catalysts is an important challenge in modern research because the employed catalysts are often very cost-effective or the obtained products are often very valuable from the economical and medicinal points of view^8–10^. Recently, heterogeneous catalysts containing an organic–inorganic material hybrid, have received much attention due to having both the advantages of homogeneous and heterogeneous catalysts^11–15^. Magnetically separable nanomaterials could be considered as a one of the most important classes of materials with unique physicochemical properties that have attracted the attention of a wide variety of researchers^16–18^. Regarding the catalytic support materials, spinel ferrite compounds have great potential in industry and technology as green heterogeneous catalysts in various organic functional group transformations and as catalytic supports^19–22^. Magnetic materials such as zinc ferrite due to their unique and non-toxic magnetic properties^23^, good biological compatibility^24^, and adjustable magnetic properties^25^, have been used in various fields including cancer therapy, drug delivery^26^, and targeting^27^. Magnetic nanoparticles have properties such as high active surface area, recyclability, chemical, and thermal stability, which are introduced as separated types of heterogeneous catalysts^28–30^. Zinc ferrite (ZnFe_2_O_4_) has the same properties as homogeneous catalysts and is also facilely extracted from the reaction by an external magnet and does not require more rigorous methods such as filtration or centrifugation. In comparison to homogeneous catalysts, heterogeneous ones have been exclusively studied because of their easy recovery and separation from the reaction mixture^31^. The use of magnetically separable catalysts is a well-favored and fascinating strategy to bridge the split between heterogeneous and homogenous catalysis processes^32^. As a main member of the ferrite family, ZnFe_2_O_4_ has promising potential for use as novel catalytic support. Additionally, the surface hydroxyl groups over them facilitate their surface modifications with a wide variety of organic compounds.

One of the most essential organic processes is the oxidation of sulfides because the corresponding sulfoxides are valuable mediators for the synthesis of chemical and biological molecules^33,34^. Furthermore, some of the sulfoxides play leading roles as therapeutic factors such as antibacterial^35^, anti-ulcer^36^, antimicrobial^37^, etc. Sulfide derivatives can be easily oxidized in the presence of a large wide variety of catalysts^38–40^. Several of these catalysts are not suitable for selective sulfoxidation reactions because of various reasons such as over oxidation to sulfones, low yields, toxicity, use of costly reagents, and low selectivity of products^41,42^.

The five-membered ring containing two Nitrogen functional groups on positions 1 and 2 generates one of the most important heterocyclic ring systems—pyrazoles. In recent years, pyrazoles and their derivatives have received great attention due to a broad spectrum of biological and pharmacological activities^43^.

Herein, we report ZnFe_2_O_4_@SiO_2_@APTES@DHBS-Cu MNPs as a green, novel, reusable, and eco-friendly nanocatalyst for the synthesis of bis (pyrazolyl)methanes derivatives and oxidation of sulfide to the sulfoxides. ZnFe_2_O_4_@SiO_2_@APTES@DHBS-Cu has a super magnetic property and thus can be simply separated from the reaction mixture using an external magnet.

## Experimental

### Materials

All required materials for the synthesis of catalyst, oxidation of sulfides, synthesis of bis (pyrazolyl) methanes, solvents, and reagents have been purchased from Fluka or Merck.

### Synthesis of ZnFe_2_O_4_

At first, for the synthesis of ZnFe_2_O_4_ magnetic nanoparticles, 2.94 g of iron (II) chloride tetrahydrate (FeCl_2_^.^4H_2_O), and 3.00 g of Zinc nitrate hexahydrate (Zn (NO_3_)_2_^.^6H_2_O) were mixed and vigorously stirred in 100 mL of deionized water. In the next step, 5 g NaOH in 50 mL deionized water was solved and added to the reaction mixture dropwise. Subsequently, the mixture reaction was stirred for 1 h under a nitrogen gas (N_2_) atmosphere. The final product was separated by a magnet, and washed three times, with hot ethanol (30 mL) and deionized water (30 mL). Finally, polycrystalline spinel magnetite nanoparticles (ZnFe_2_O_4_) were dried at 65 °C^44^.

### Preparation of nano-ZnFe_2_O_4_@SiO_2_ core shells

In the second step, 1.0 g of the obtained ZnFe_2_O_4_ was dispersed in a mixture of EtOH (50 mL), 5.0 mL of ammonia solution, 10 mL of H_2_O, followed by the addition of 2.65 g of Polyethylene glycol (PEG-400) and 3 mL of tetraethyl orthosilicate (TEOS). After that, the reaction mixture was stirred at 25 °C for 34 h. Eventually, the product was separated by a magnet and washed with EtOH (30 mL) and deionized water (30 mL) five times, and dried at 25 °C.

### Preparation of ZnFe_2_O_4_@SiO_***2***_@APTES@DHBS-Cu(I)

**Figure 1:**
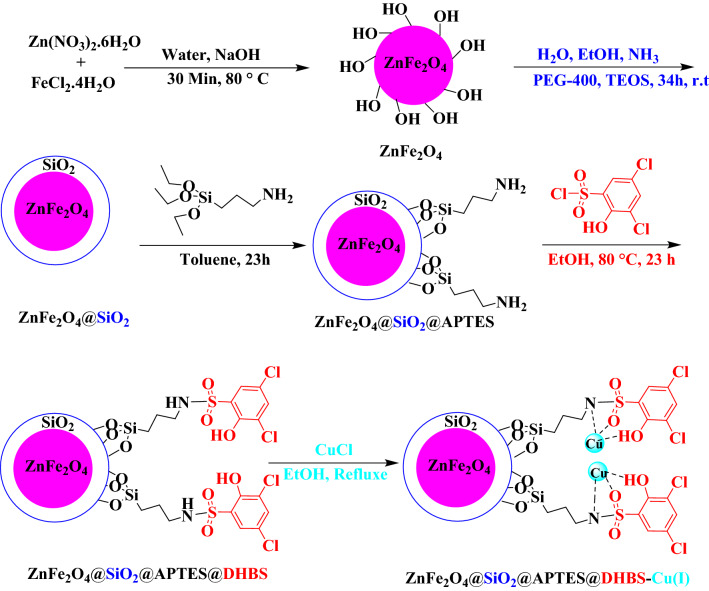
Synthesis of ZnFe_2_O_4_@SiO_2_@APTES@DHBS-Cu.

The novel ZnFe_2_O_4_@SiO_2_@APTES@DHBS-Cu MNPs were readily synthesized according to the route depicted in (Fig. 1). In the next step, ZnFe_2_O_4_@SiO_2_@APTES@DHBS-Cu magnetic nanoparticles were synthesized using the following steps. First for functionalization of ZnFe_2_O_4_@SiO_2_ by 3-(Triethoxysilyl)propylamine, 1 g obtained ZnFe_2_O_4_@SiO_2_ nanoparticles were added to 30 mL of a toluene solution and then 2 mL of 3-(Triethoxysilyl)propylamine was added dropwise to this mixture. The reaction mixture was stirred at reflux condition for 23 h. Then, the resulting solid was filtered, washed with ethanol (30 mL) and water (30 mL) several times, and dried at room temperature. An appropriate amount of ZnFe_2_O_4_@SiO_2_@APTES (1 g) was dispersed in deionized water (100 mL) by sonication for 20 min. Subsequently, 2.5 mmol of 3,5-dichloro-2-hydroxybenzenesulfonyl chloride (DHBS) was added and the reaction mixture was stirred at 85 °C for 23 h. The reaction was performed under a nitrogen gas (N_2_) atmosphere. Finally, to prepare ZnFe_2_O_4_@SiO_2_@APTES@DHBS-Cu, the obtained ZnFe_2_O_4_@SiO_2_@APTES@DHBS (1 g) was dispersed in 30 mL by EtOH sonication for 20 min. Subsequently, 2.5 mmol of Copper (I) chloride was added to the reaction mixture which was stirred under the nitrogen gas (N_2_) atmosphere at reflux conditions (90 °C) for 24 h a day. Afterward, the reaction mixture was cooled at 25 °C and, then the final ZnFe_2_O_4_@SiO_2_@APTES@DHBS-Cu (MNPs) were isolated, using a magnet, from the reaction mixture, washed by EtOH (30 mL) and distilled water (30 mL) (several times), to remove the residual impurities and, eventually, and then dried at 75 °C for 20 h^45^.

### General procedure for the synthesis of bis (pyrazolyl) methanes

**Figure 2:**
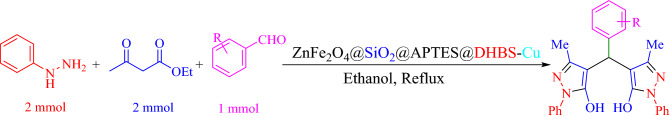
preparation of bis (pyrazolyl)methanes.

In a round bottom balloon, a mixture of phenylhydrazine (2 mmol), ethyl acetoacetate (2 mmol), and substituted aromatic aldehydes (1 mmol) and ZnFe_2_O_4_@SiO_2_@APTES@DHBS-Cu (0.03 g) was added, and the reaction mixture was refluxed in ethanol for 60 min. Completion of the following reaction has been analyzed via TLC. After the reaction mixture was diluted with hot ethanol to dissolve the organic products, the catalyst was separated using an external magnet and the resultant unrefined bis (pyrazolyl) methane products, were further purified through recrystallization in an ethanol (Fig. 2).

### A general procedure for the oxidation of sulfides

**Figure 3:**
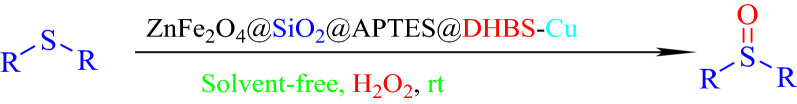
Oxidation of sulfides to sulfoxides catalyzed by ZnFe_2_O_4_@SiO_2_@APTES@DHBS-Cu.

A combination of sulfide (2 mmol) and H_2_O_2_ 33% (0.3 mL) was poured into the round-bottomed flask containing ZnFe_2_O_4_@SiO_2_@APTES@DHBS-Cu MNPs (0.02 g). The resulting mixture was stirred under solvent-free conditions at 25 °C. After the completion of the reaction, the ZnFe_2_O_4_@SiO_2_@APTES@DHBS-Cu MNPs were separated by a magnet, and the products were extracted with water and ethyl acetate. Finally, the combined organics were dried over anhydrous sodium sulfate (2 g). Evaporation of the solvent gave the pure corresponding sulfoxides an excellent yield (Fig. 3).

### Selected NMR data

- Sulfinyldibenzene: ^1^H NMR (250 MHz, CDCl_3_): 7.22–7.65 (m, 10H) ppm.
- Methylsulfinyl)benzene: ^1^H NMR (250 MHz, CDCl_3_):2.7 (s, 3H), 7.48–7.93 (m, 5H) ppm.
- 1-(Butylsulfinyl)butane: ^1^H NMR (250 MHz, CDCl_3_): 0.94(t, J = 7.5 Hz, 6H), 1.40 (m, 4H), 1.70 (m, 4H), 2.66 (t, J = 7.5 Hz, 4H) ppm.
- 4,4′-((4-Tolyl)methylene)bis(3-methyl-1-phenyl-1H-pyrazol-5-ol): 2.29 (s, 6H), 2.41 (s, 3H), 4.79 (s, 1H), 7.09–7.69 (m, 14H), 7.72 (s, 2H) ppm.
- 4,4′-((4-Methoxyphenyl)methylene)bis(3-methyl-1-phenyl-1H-pyrazol-5-ol): ^1^H NMR (250 MHz, CDCl_3_): *δ* 2.25 (s, 6H), 3.75 (s, 3H), 4.77 (s, 1H), 6.77–7.95 (m, 14H), 8.57–8.61 (b, 2H) ppm.

## Result and discussion

### Catalyst characterization

**Figure 4:**
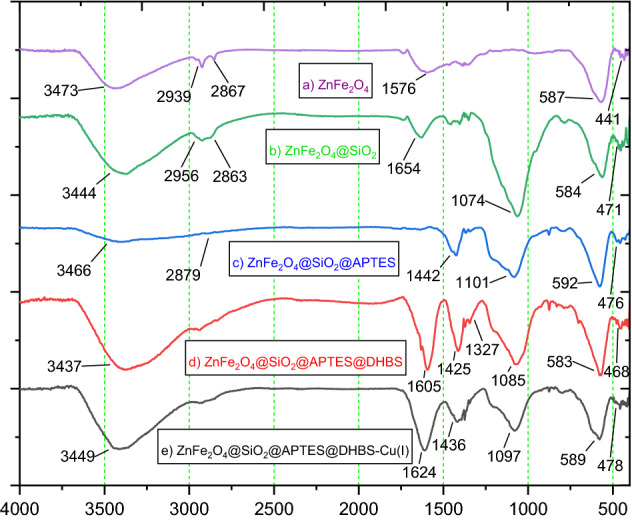
FT-IR spectra of (**a**) ZnFe_2_O_4_, (**b**) ZnFe_2_O_4_@SiO_2_, (**c**) ZnFe_2_O_4_@SiO_2_@APTES, (**d**) ZnFe_2_O_4_@SiO_2_@APTES@DHBS, (**e**) ZnFe_2_O_4_@SiO_2_@APTES@DHBS-Cu.

The FT-IR spectra of the (a) ZnFe_2_O_4_, (b) ZnFe_2_O_4_@SiO_2_, (c) ZnFe_2_O_4_@SiO_2_@APTES, (d) ZnFe_2_O_4_@SiO_2_@ APTES@DHBS and (e) ZnFe_2_O_4_@SiO_2_@APTES@DHBS-Cu (catalyst) are presented in Fig. 4. Using FT-IR spectroscopy the synthesis of zinc ferrite nanoparticles (ZF-NPS) was confirmed. The two absorption bands at 441 and 587 cm^−1^ are assigned to the stretching vibrations of the zinc–oxygen and the iron-oxygen bonds, respectively. In Fig. 4a, the bending and stretching vibration of adsorbed water molecules on the surface of the nanoparticles at 1576 and 3473 cm^−1^ are respectively assigned^46^. Figure 4b confirms the condensation reaction between hydroxyl groups of ZnFe_2_O_4_ (MNPs) and the alkoxysilane molecules of TEOS as the first layer. Absorbed peaks at 3444 and 2956 cm^−1^ were specified as hydroxide stretching vibration mode. The three absorption peaks around 1074, 584 cm^−1^, and 471 cm^−1^ indicated the presence of Si–O–Si asymmetric and symmetric stretching vibrations and bending vibration mode of Si–O–Si, as well as a small peak around 1658 cm^−1^, was assigned to hydroxide stretching vibration of Silicon-hydroxy and twisting vibration of adsorbed H–O–H in a silica shell^47^. In Fig. 4c, In ZnFe_2_O_4_@SiO_2_@ APTES, the bands in the range of 2879 cm^−1^ correspond to the bending vibration of CH_2_ confirming the attachment of APTES chain molecules^48,49^. The bands at 1442 cm^−1^ correspond to NH due to the deformation of the hydrogen-bonded amine groups respectively. NH_2_ stretching vibrations are present at^50,51^ 3466 cm^−1^. In the spectra of ZnFe_2_O_4_@SiO_2_@APTES@DHBS (d) the peak in the 1327 cm^−1^ is attributed to the appearance of sulfone groups (–SO_2_–) and the peaks in the 1425 and 1605 cm^−1^ correspond to the carbon–carbon double bond (C=C) in the aromatic ring. Moreover, a certain band in the 3437 cm^−1^ is due to aromatic O–H^45^. In Fig. 4e, the spectrum FT-IR shows no significant changes in the ZnFe_2_O_4_@SiO_2_@APTES@DHBS-Cu MNPs vibration bands, and only a slight difference in the intensity of bands can be noticed. These observations demonstrate the immobilization of Cu on the surface of ZnFe_2_O_4_@SiO_2_@APTES@DHBS MNPs (Fig. 4e).

**Figure 5:**
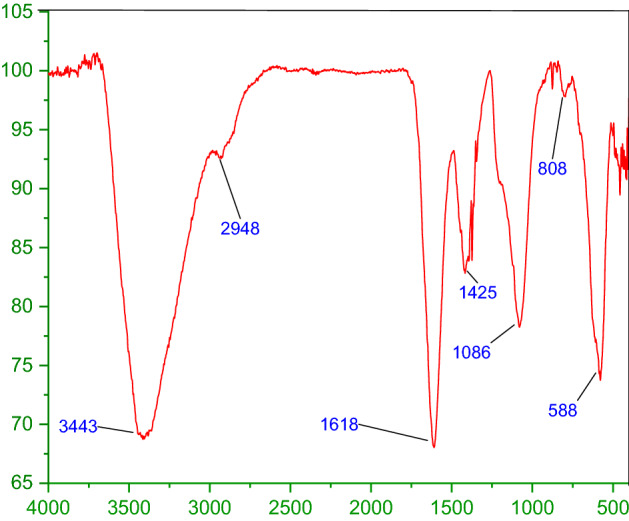
FT-IR analysis of recovered ZnFe_2_O_4_@SiO_2_@APTES@DHBS-Cu.

A comparison of FT-IR spectra of the catalyst after recycling is shown in Fig. 5. As shown in this figure, there are no changes in the FT-IR of ZnFe_2_O_4_@SiO_2_@APTES@DHBS-Cu after recovery, which confirmed the stability of this catalyst under reaction conditions.

**Figure 6:**
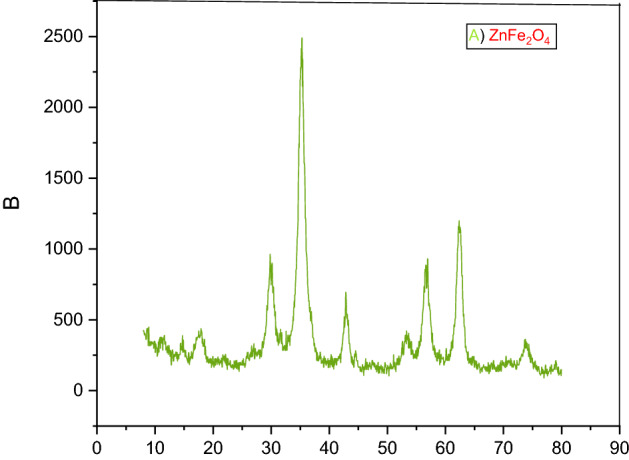
XRD spectrum of ZnFe_2_O_4_ (**A**).

**Figure 7:**
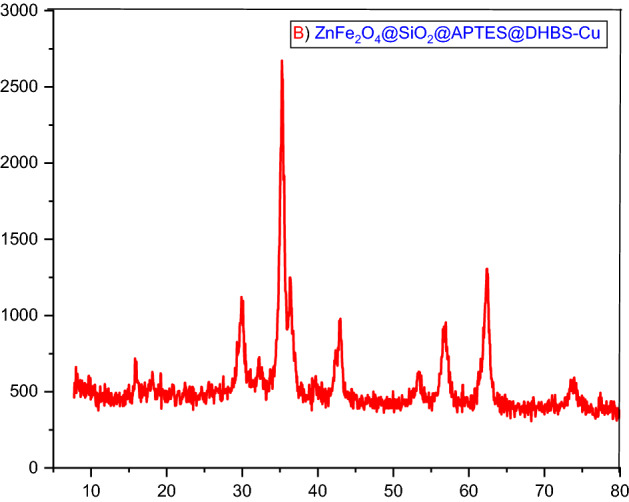
XRD spectrum of ZnFe_2_O_4_@SiO_2_@APTES@DHBS-Cu (**B**).

X-ray diffraction (XRD) analysis was used to investigate and identify the structure of the zinc ferrite sample. XRD patterns of ZnFe_2_O_4_ and ZnFe_2_O_4_@SiO_2_@APTES@DHBS-Cu samples are shown in Figs. 6 and 7. Several strong characteristic peaks are seen in the area of 2θ = 8–80. According to X-ray diffraction peaks at 2θ = 18.2°, 30.5°, 35.7°, 43.4°, 57.4°, 62.9°, 74.6° from ZnFe_2_O_4_@SiO_2_@APTES@DHBS-Cu NPS confirm that the particles have a crystalline structure in the cubic spinel phase^52–54^.

**Figure 8:**
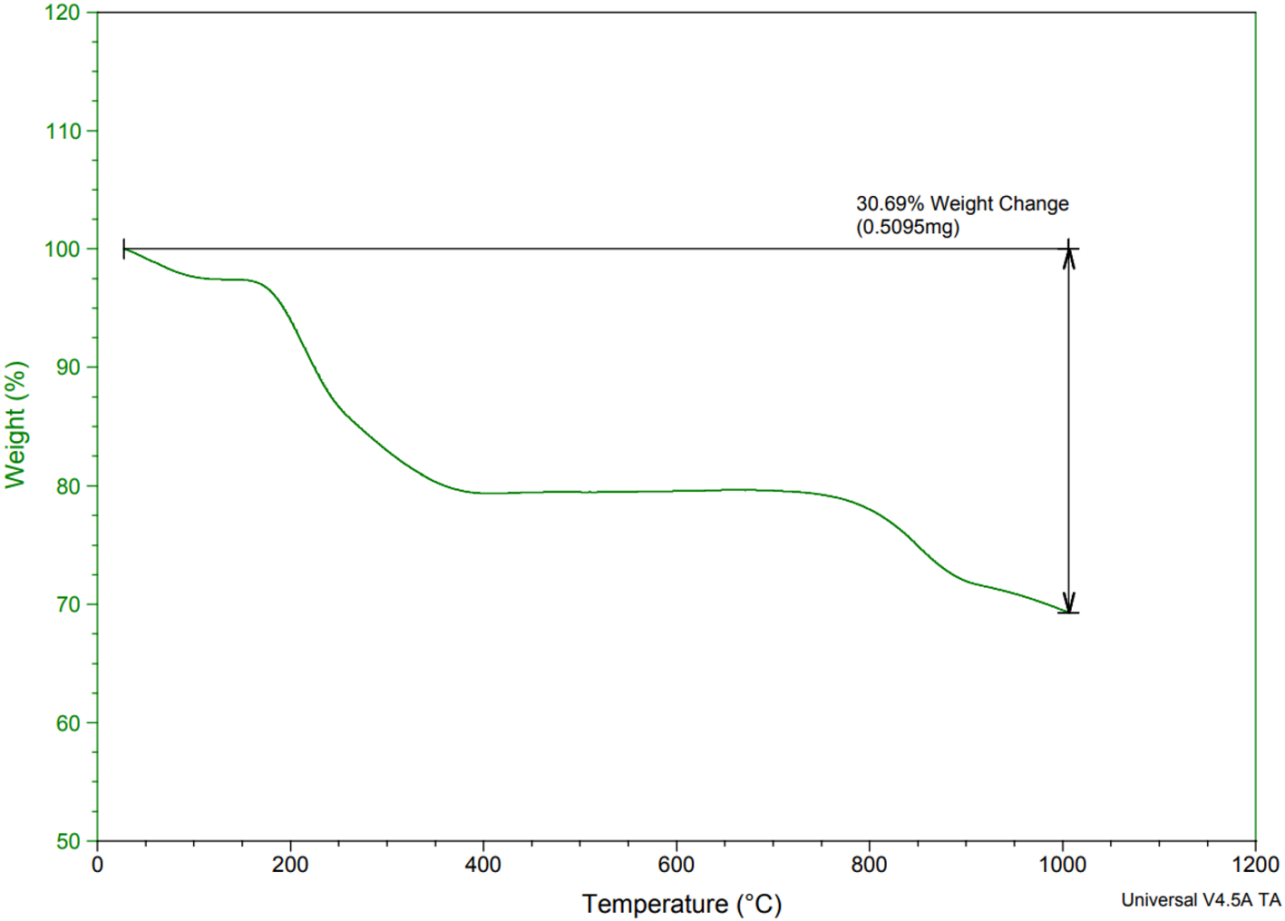
The TGA curves of a ZnFe_2_O_4_@SiO_2_@APTES@DHBS-Cu.

The TGA results of ZnFe_2_O_4_@SiO_2_@APTES@DHBS-Cu are shown in Fig. 8. Thermal stability and the presence of organic matter fixed on MNPs were investigated using TGA. According to the curve of ZnFe_2_O_4_@SiO_2_@APTES@DHBS-Cu, the weight loss of about 3% below 200 °C is due to the removal of physically adsorbed water and surface hydroxyl groups. In addition, according to the TGA curves, observed the weight loss was about 12% at 200–650 °C for ZnFe_2_O_4_@SiO_2_@APTES@DHBS-Cu that is contributed to the thermal decomposition of immobilized organic components on the ZnFe_2_O_4_ surface^45^.

**Figure 9:**
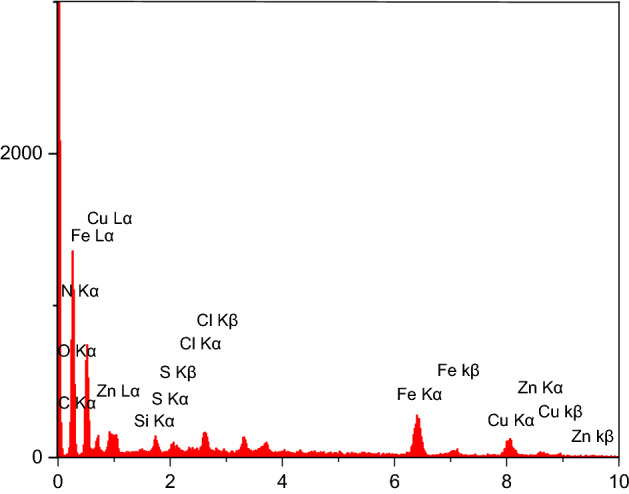
EDS spectrum of ZnFe_2_O_4_@SiO_2_@APTES@DHBS-Cu.

The EDX analysis is brought in Fig. 9 for ZnFe_2_O_4_@SiO_2_@APTES@DHBS-Cu. In another investigation, EDX analysis confirmed the existence of silicon, oxygen, chlorine, sulfur, Iron, Carbon, Nitrogen, and Copper elements in the synthesized magnetic nanoparticles and confirm the synthesis of ZnFe_2_O_4_@SiO_2_@APTES@DHBS-Cu. The existence of Copper peaks in this spectrum indicates the loading of Cu onto the ZnFe_2_O_4_@SiO_2_@APTES@DHBS.

**Figure 10:**
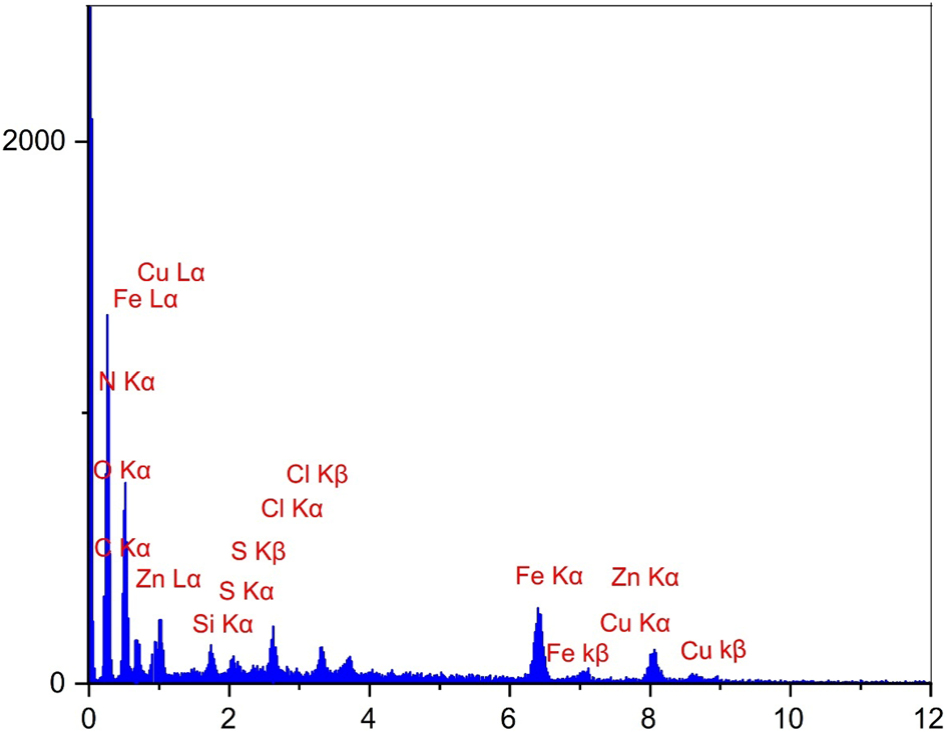
EDS spectrum of recovered ZnFe_2_O_4_@SiO_2_@APTES@DHBS-Cu.

Figure 10 shows the comparison of the EDS spectrum of the catalyst after recycling. As shown, there are no changes in the EDS spectrum of ZnFe_2_O_4_@SiO_2_@APTES@DHBS-Cu after recovery, which confirmed the stability of this catalyst under optimal reaction conditions.

**Figure 11:**
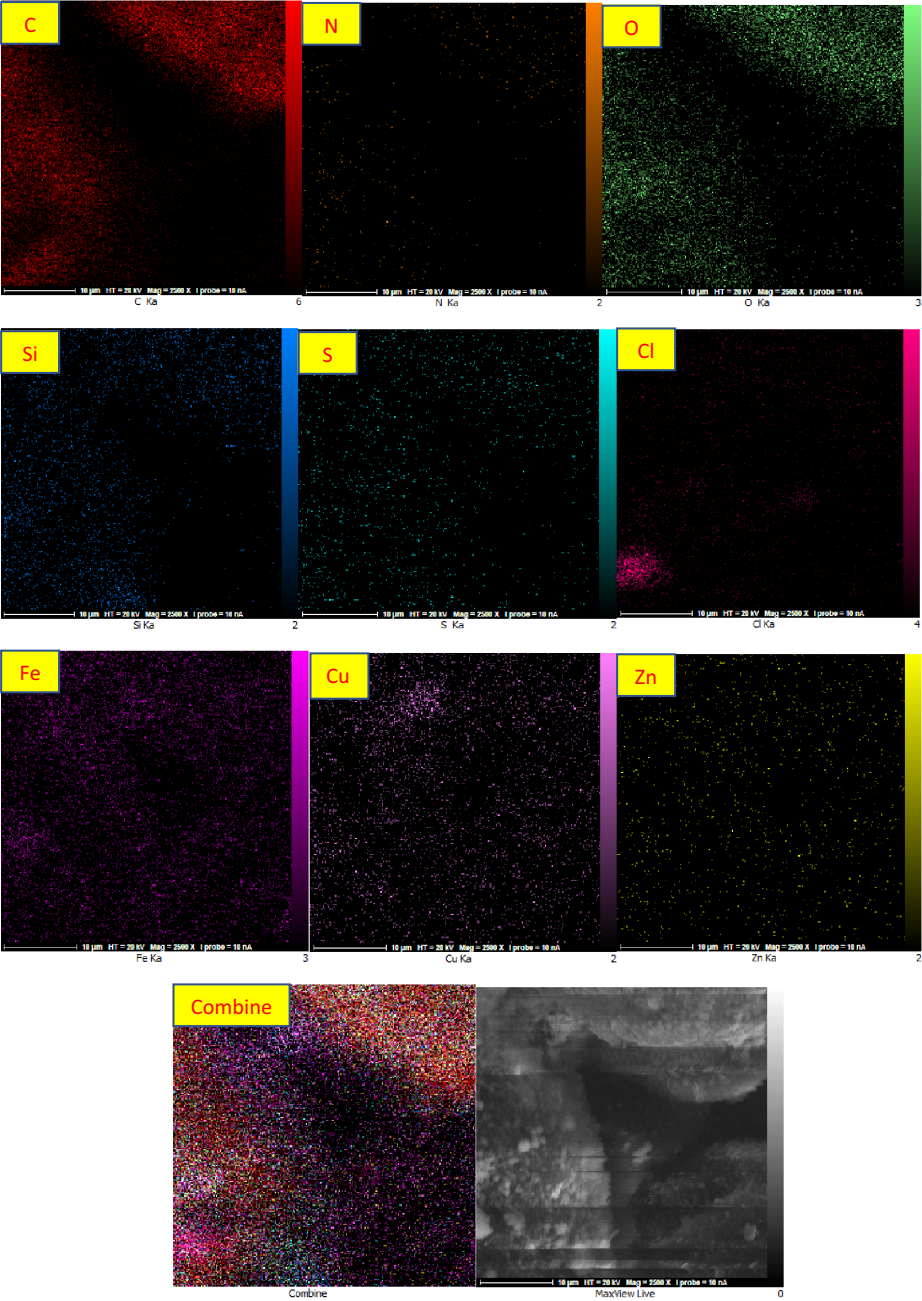
X-ray map spectrum of ZnFe_2_O_4_@SiO_2_@APTES@DHBS-Cu.

The X-ray mapping analysis was done to determine the element's contents of ZnFe_2_O_4_@SiO_2_@APTES@DHBS-Cu and their distribution (Fig. 11). The ordered dispensation of the elements (Zn, C, S, Si, Fe, O, N, Cl, and Cu) was observed in the nanocatalyst structure. Thus the conclusion is reached that the Copper has uniformly dispersed on the surface of ZnFe_2_O_4_@SiO_2_@APTES@DHBS.

**Figure 12:**
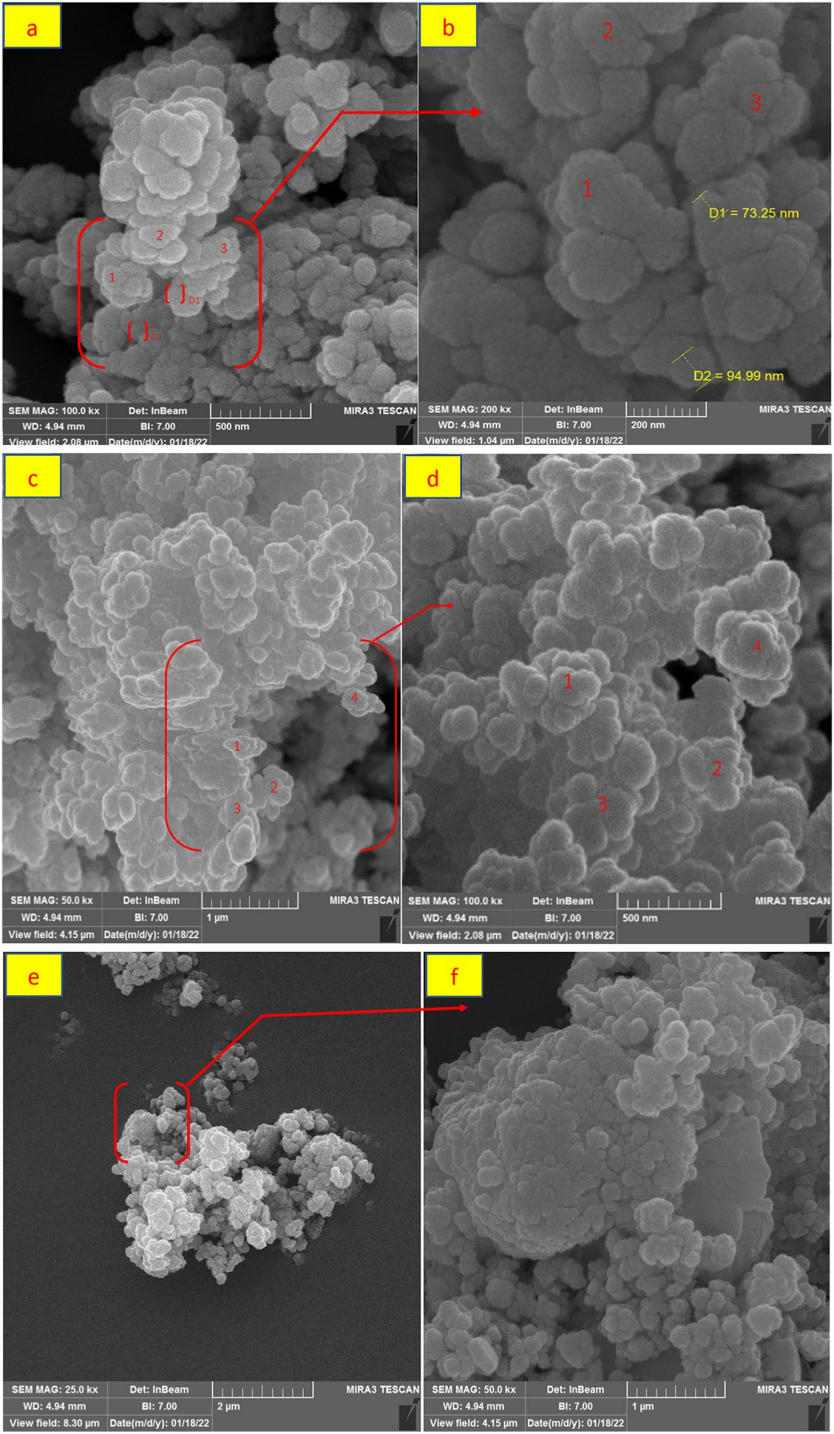
SEM spectrum of ZnFe_2_O_4_@SiO_2_@APTES@DHBS-Cu.

Figure 12a–f shows the high-magnification SEM images of the ZnFe_2_O_4_@SiO_2_@APTES@DHBS-Cu MNPs. Based on these images, The ZnFe_2_O_4_@SiO_2_@APTES@DHBS-Cu magnetic nanoparticle is spherical with an almost homogenous size distribution. Furthermore, It is found that ZnFe_2_O_4_@SiO_2_@APTES@DHBS-Cu magnetic nanoparticles are about 73–94 nm in size (Fig. 12b).

**Figure 13:**
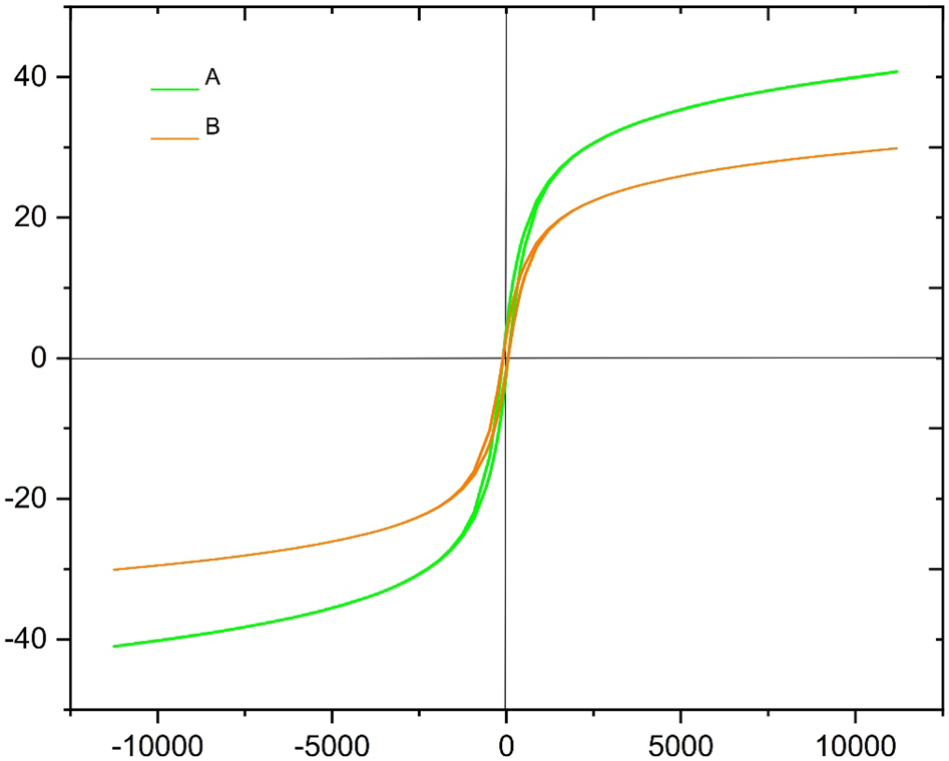
The magnetic properties of (**a**) ZnFe_2_O_4_ and (**b**) ZnFe_2_O_4_@SiO_2_@APTES@DHBS-Cu.

The VSM pattern of ZnFe_2_O_4_@SiO_2_@APTES@DHBS-Cu and ZnFe_2_O_4_ magnetic was shown in Fig. 13. The magnetic values were 41.5 and 31 emu/g for ZnFe_2_O_4_ and ZnFe_2_O_4_@SiO_2_@APTES@DHBS-Cu, respectively. Due to the coating of nanoparticles with silica and DHBS-Cu, the magnetic properties value of the final catalyst decreased compared to the bare ZnFe_2_O_4_. Due to the relatively high magnetic properties of the prepared nanomaterial, the particles were effectively recycled and removed from the mixture.

### Catalytic study

#### Catalytic activity studies of ZnFe_2_O_4_@SiO_2_@APTES@DHBS-Cu for the synthesis of bis(pyrazolyl) methanes via the pseudo-five-component reaction

In primary research to obtain optimal reaction conditions, the effect of temperature, solvent type, and catalyst quantity outcome of the reaction was checked on the condensation of phenylhydrazine (2 mmol), ethyl acetoacetate (2 mmol), and 4-chloro benzaldehyde (1 mmol). First, the effect of solvents was considered. Surprisingly, EtOH (2 mL) was the most suitable solvent to reach the desired product (Table 1). Then, the effects of reaction temperature were evaluated on the model reaction. Among the various screened temperatures (e.g., 25, 50, 70, 100 °C and reflux conditions), the reflux conditions were the most effective temperature for described reaction (Table 1, entry 5). Then, the influence of the catalyst loading on the outcome of the reaction was studied with different amounts of catalyst. Obtained results revealed that 0.03 g of the catalyst is the most effective amount (Table 1, entry 5). As given in Table 1, entry 1, the reaction was not completed in the absence of the catalyst even after 8 h. Eventually, EtOH at reflux condition in the presence of 0.03 g of ZnFe_2_O_4_@SiO_2_@APTES@DHBS-Cu MNPs was found to be the best choice for the described multicomponent reaction. For indicating the exigency of the presence of Cu metal in the nanocatalyst, the model reaction was undertaken in the presence of ZnFe_2_O_4_ and ZnFe_2_O_4_@SiO_2_ instead of ZnFe_2_O_4_@SiO_2_@APTES@DHBS-Cu (Table 1, entries16 and 17) that surprisingly no any product was produced.

**Table 1:** The results of optimizing the catalyst quantity, temperature, and solvent on the reaction of 4-chlorobenzaldehyde, phenylhydrazine, and ethyl acetoacetate.

Entry	Catalyst (g)	Solvent	Temperature (°C)	Time (min)	Yield
1	–	Ethanol	Reflux	8 h	Trace
2	0.005	Ethanol	Reflux	60	65
3	0.01	Ethanol	Reflux	60	87
4	0.02	Ethanol	Reflux	60	93
5	0.03	Ethanol	Reflux	60	96
6	0.04	Ethanol	Reflux	60	96
7	0.03	Acetonitrile	Reflux	60	65
8	0.03	H_2_O	Reflux	60	NR
9	0.03	Dimethyl sulfoxide	100	60	87
10	0.03	PEG-400	100	60	70
11	0.03	Dimethyl formamide	100	60	81
12	0.03	Solvent free	100	60	59
13	0.03	Ethanol	R. T	60	NR
14	0.03	Ethanol	50	60	58
15	0.03	Ethanol	70	60	89
16	0.03^a^	Ethanol	Reflux	60	N. R
17	0.03^b^	Ethanol	Reflux	60	N. R

^a^Reaction was performed in the presence of ZnFe_2_O_4_ NPs.

^b^Reaction was performed in the presence of nano-ZnFe_2_O_4_@SiO_2_.

After optimization, we examined various electron-withdrawing and electron-releasing substituted benzaldehydes in ZnFe_2_O_4_@SiO_2_@APTES@DHBS-Cu catalyzed multicomponent cyclo condensation reaction for the preparation of bis(pyrazolyl) methane derivatives to identify the generality and the high proficiency of catalytic system (Table 2). It is evident from Table 2 that a variety of bis(pyrazolyl) methane derivatives were synthesized with values of melting point, yield, and reaction time. As shown in Table 2, this catalytic system is a suitable method for the efficiency of conditions. All derivatives were obtained with excellent yields (70–96%) and short reaction times (45–95 min). Also, the reaction with electron-withdrawing benzaldehydes (NO_2_, Cl, and Br) is considered faster than the one with electron-donating benzaldehydes (Me, OMe, and OH).

**Table 2:** The one-pot pseudo-five-component production of bis (pyrazolyl)methanes catalyzed by ZnFe_2_O_4_@SiO_2_@APTES@DHBS-Cu.

Entry	Ar	Product	Time (min)	Yield (%)^b^	TON	TOF (h^−1^)	M.P. (°C)	
							Measured	Literature
1	C_6_H_5_		45	92	102	136	169–176	171–177^55^
2	4-ClC_6_H_5_		60	96	106	106	214–217	213–218^55^
3	2,6-ClC_6_H_5_		50	70	77.7	97	247–251	248–253^56^
4	2,4-ClC_6_H_5_		45	84	93.3	124	220–226	223–227^57^
5	2-BrC_6_H_5_		60	92	102	102	197–205	198–206^55^
6	4-MeC_6_H_5_		60	93	155	155	200–207	203–209^55^
7	4-MeOC_6_H_5_		65	89	98.8	91.4	170–178	173–179^55^
8	4-OHC_6_H_5_		55	91	101	112	151–158	153–158^55^
9	3,4-MeOHC_6_H_5_		95	91	101	67	192–195	193–197^55^
10	2-NO_2_C_6_H_5_		45	88	97.7	129	223–227	225–228^55^
11	3-NO_2_C_6_H_5_		70	85	94.4	85.4	150–154	151–154^55^

^a^Isolated yield.

^b^Reaction conditions: phenylhydrazine (2 mmol), ethyl acetoacetate (2 mmol), aromatic aldehydes (1 mmol), ZnFe_2_O_4_@SiO_2_@APTES@DHBS-Cu in EtOH (2 mL) under reflux conditions.

**Figure 14:**
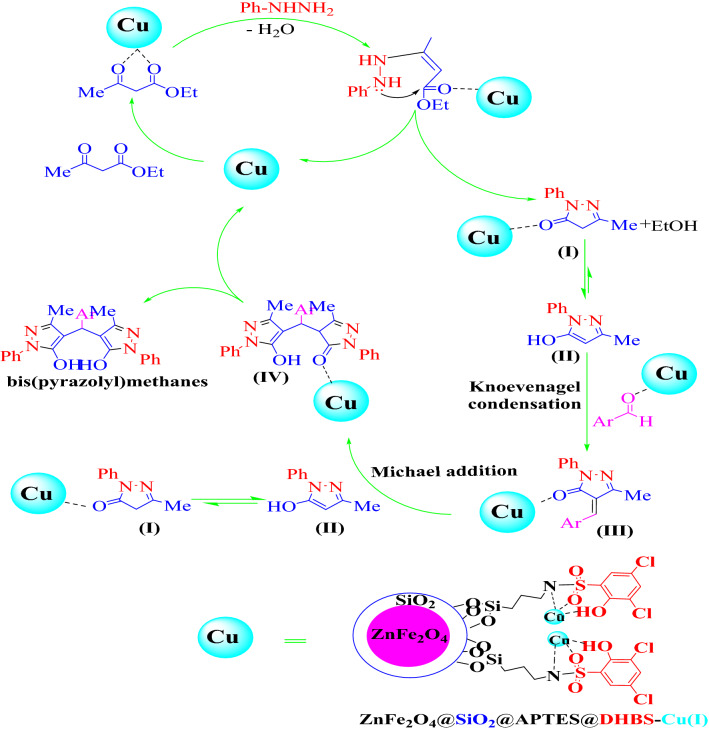
The possible mechanism for ZnFe_2_O_4_@SiO_2_@APTES@DHBS-Cu catalyzed the pseudo-five-component preparation of bis (pyrazolyl) methanes.

Based on our previous studies, a proposed possible mechanism for synthesis of bis (pyrazolyl)methanes using ZnFe_2_O_4_@SiO_2_@APTES@DHBS-Cu nanocatalyst has been presented in Fig. 14. Initially, the carbonyl group in the ethyl acetoacetate was activated by the ZnFe_2_O_4_@SiO_2_@APTES@DHBS-Cu nanocatalyst for the attack of lone pair of nitrogen form phenylhydrazine to form intermediate pyrazolone (I). In the next step, the activated aromatic aldehyde by ZnFe_2_O_4_@SiO_2_@APTES@DHBS-Cu nanocatalyst undergoes a tandem reaction with intermediate (II) (which is the tautomer of intermediate (I)) leading to intermediate (III) after removal of an H_2_O molecule. The next step is a Michael addition of another intermediate of (III) to (II) to form intermediate (IV). In the last step, after the tautomeric proton shift, afford bis(pyrazolyl)methanes.

In another part of this research project, the catalytic activity of ZnFe_2_O_4_@SiO_2_@APTES@DHBS-Cu was tested in the oxidation of sulfide. The reaction of the methyl phenyl sulfide with H_2_O_2_ (as a green oxidizing agent) was chosen as a model reaction (for optimization). The effect of various solvents with different polarities (including Ethyl acetate, Ethanol, n-Hexane, EtOH: H_2_O (1:1), H_2_O) as well as solvent-free conditions, was checked. The best reaction yield was gained under solvent-free conditions at 30 min (Table 3, Entry 5). Then the amount of catalyst was optimized. The results of experiments indicated oxidation reaction didn’t occur in the absence of ZnFe_2_O_4_@SiO_2_@APTES@DHBS-Cu even after 360 min (Table 3, entry 1). The details of reaction parameters and results are brought in in Table 3. As shown in Table 3, (entries 16 and 17), the reaction was performed in the presence of ZnFe_2_O_4_ and ZnFe_2_O_4_@SiO_2_@APTES@DHBS (0.02 g) for the oxidation of sulfides. Meanwhile, the best yield of products was obtained in the presence of ZnFe_2_O_4_@SiO_2_@APTES@DHBS-Cu (0.02 g).

**Table 3:** Optimizing reaction conditions for oxidation of methyl phenyl sulfide in the presence of ZnFe_2_O_4_@SiO_2_@APTES@DHBS-Cu.

Entry^a^	Catalyst (g)	Solvent	H_2_O_2_ (mg)	Time (min)	Yield (%)^b^
1	–	Solvent-free	0.3	360	Trace
2	0.003	Solvent-free	0.3	30	75
3	0.007	Solvent-free	0.3	30	85
4	0.01	Solvent-free	0.3	30	87
5	0.02	Solvent-free	0.3	30	97
6	0.03	Solvent-free	0.3	30	97
7	0.02	*n*-Hexane	0.3	30	58
8	0.02	H_2_O	0.3	30	Trace
9	0.02	H_2_O: EtOH	0.3	30	47
10	0.02	EtOAc	0.3	30	92
11	0.02	EtOH	0.3	30	82
12	0.02	Solvent-free	0.1	30	88
13	0.02	Solvent-free	0.2	30	92
14	0.02	Solvent-free	0.3	30	96
15	0.02	Solvent-free	0.4	30	96
16	0.02	Solvent-free	0.3	30	N. R^c^
17	0.02	Solvent-free	0.3	30	Trace^d^

^a^Reaction conditions: sulfide (1 mmol) H_2_O_2_ (0.3 mL) and ZnFe_2_O_4_@SiO_2_@APTES@DHBS-Cu at 25 °C under solvent-free conditions.

^b^Isolated yield.

^c^The reaction was catalyzed by ZnFe_2_O_4_.

^d^The reaction catalyzed by ZnFe_2_O_4_@SiO_2_@APTES@DHBS.

After the optimization, the oxidation of different sulfides was investigated in the presence of ZnFe_2_O_4_@SiO_2_@APTES@DHBS-Cu NPs. In all cases, sulfoxides were produced in high yields at short reaction times, which showed efficiency and excellent catalytic activity of described catalyst. As shown in Table 4, this catalytic system is a suitable method in terms of the efficiency of conditions. All derivatives were obtained with excellent yields and short reaction times. Also, the reaction with electron-withdrawing benzaldehydes is considered faster than the one with electron-donating benzaldehydes. The results including product yields and reaction times obtained are summarized in Table 4.

**Table 4:** Oxidation of sulfides into sulfoxides in the presence of ZnFe_2_O_4_@SiO_2_@APTES@DHBS-Cu.

Entry^a^	Substrate	Time (min)	Yield (%)^b^	TON	TOF (h^−1^)	Melting point (°C)	
						Measured	Literature
1		35	96	160	275	31–35	32–36^58^
2		30	95	158	316	130–132	128–131^58^
3		90	75	125	83.3	151–156	150–154^59^
4		75	87	145	116	85–89	83–86^60^
5		70	90	150	129	Oil	Oil
6		35	86	143	246	Oil	Oil
7		45	90	150	200	116–120	114–116^61^
8		35	93	155	267	Oil	Oil
9		85	72	120	85.1	67–68	70–72^58^

^a^Reaction conditions: sulfide (1 mmol) H_2_O_2_ (0.3 mL) and ZnFe_2_O_4_@SiO_2_@APTES@DHBS-Cu (0.02 g) at room temperature under solvent-free conditions.

^b^Isolated yield.

**Figure 15:**
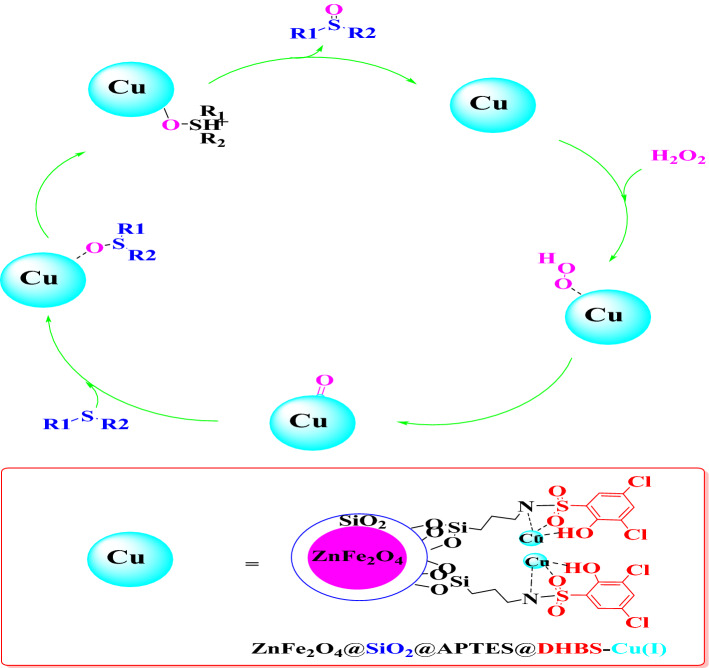
The suggested mechanism for the oxidation of sulfide.

Based on previous studies, a suggested and possible mechanism for the sulfoxidation reaction catalyzed by ZnFe_2_O_4_@SiO_2_@APTES@DHBS-Cu has been presented in Fig. 15. The role of copper in ZnFe_2_O_4_@SiO_2_@APTES@DHBS-Cu as a magnetic nanocatalyst is to form the active oxidant complex. Based on this mechanism, the transfer of oxygen to sulfur leads to the formation of sulfoxide.

### Hot filtration

To approve the heterogeneous nature of the ZnFe_2_O_4_@SiO_2_@APTES@DHBS-Cu in the synthesis of bis (pyrazolyl) methane compounds hot filtration experiment was performed using 4-chlorobenzaldehyde as a model substrate under the optimal reaction conditions. After half time of the reaction (30 min), the reaction was terminated, and the yield was found to be 53%. In another experiment, the reaction was designed again, and at half time of reaction, the catalyst was separated and the reaction mixture was stirred further without the catalyst. Herein, we observed that low conversion (< 4%) of the product happened through the heating of the catalyst-free mixture for another 30 min. It can be concluded that nanocatalyst is completely heterogeneous.

Also, copper leaching of ZnFe_2_O_4_@SiO_2_@APTES@DHBS-Cu was studied by atomic absorption spectroscopy (AAS). Based on AAS analysis, the amount of copper in fresh and reused catalysts were 3.6 × 10^–4^ mol g^−1^ and 3 × 10^–4^ mol g^−1^ respectively, which shows that Cu leaching into reaction media from the ZnFe_2_O_4_@SiO_2_@APTES@DHBS-Cu framework is very low.

### Reusability of catalyst

**Figure 16:**
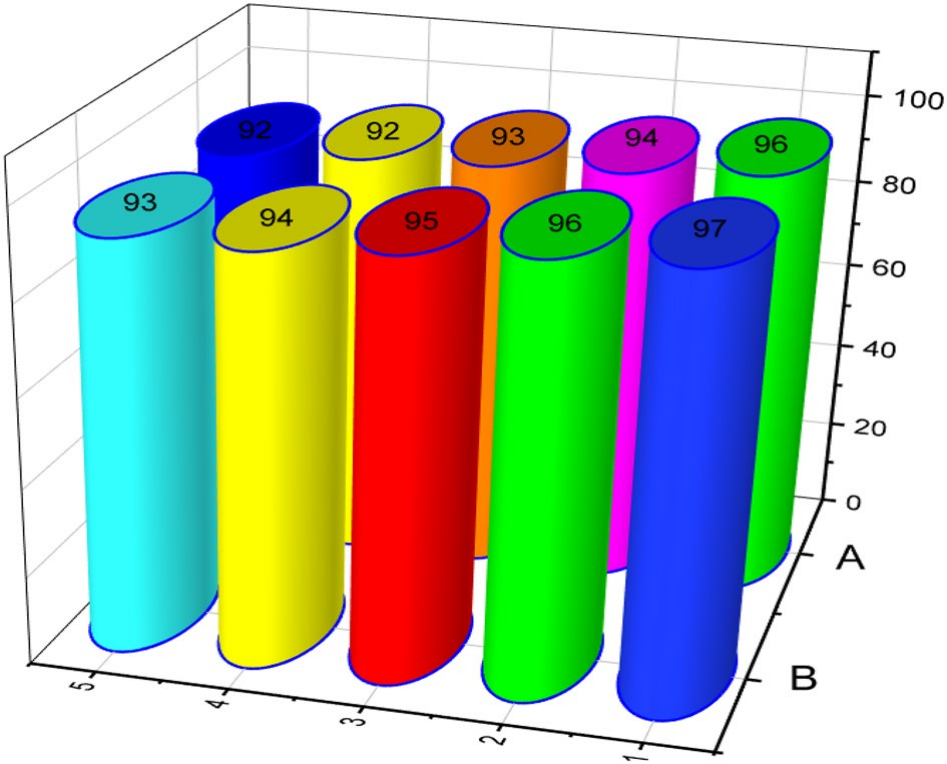
Reusability of ZnFe_2_O_4_@SiO_2_@APTES@DHBS-Cu in the oxidation of sulfides (**A**) and synthesis of bis (pyrazolyl)methanes (**B**).

Easy separation of a catalyst is an important point of view in heterogeneous catalysts. Therefore, we next considered the reusability of ZnFe_2_O_4_@SiO_2_@APTES@DHBS-Cu in the oxidation of sulfides (A) and synthesis of bis (pyrazolyl)methanes (B) using methyl phenyl sulfide and 4-chlorobenzaldehyde respectively as a model substrates. These reactions were performed under optimized conditions to test the reusability of the ZnFe_2_O_4_@SiO_2_@APTES@DHBS-Cu (Fig. 16). As can be seen from Fig. 16, no significant decrease in the yield of products was observed after five runs. Naturally, ZnFe_2_O_4_@SiO_2_@APTES@DHBS-Cu showed excellent performance in several successive cycles for each of the above reactions.

### Comparison of the catalyst

The efficiency of ZnFe_2_O_4_@SiO_2_@APTES@DHBS-Cu was investigated by comparison of our results on the synthesis of bis (pyrazolyl)methanes and oxidation of sulfides in model reactions with the previously reported methods (Table 5). The products were obtained in higher yields over faster times in the presence of ZnFe_2_O_4_@SiO_2_@APTES@DHBS-Cu. Also, this catalyst has several advantages in terms of non-toxicity, price, stability, and easy separation.

**Table 5:** Comparison results of ZnFe_2_O_4_@SiO_2_@APTES@DHBS-Cu with other catalysts in the synthesis of bis (pyrazolyl)methanes and oxidation of sulfides.

Entry	Product	Catalyst	Time (min)	Yield (%)	Refs.
1	4,4′-((4-Chlorophenyl)methylene)bis(3-methyl-1-phenyl-1H-pyrazol-5-ol)	Fe_3_O_4_@THAM-Pd	55	65	^62^
2	4,4′-((4-Chlorophenyl)methylene)bis(3-methyl-1-phenyl-1H-pyrazol-5-ol)	DCDBTSD	49	80	^63^
3	4,4′-((4-Chlorophenyl)methylene)bis(3-methyl-1-phenyl-1H-pyrazol-5-ol)	[TMEDSA][HSO_4_]_2_	30	93	^64^
4	4,4′-((4-Chlorophenyl)methylene)bis(3-methyl-1-phenyl-1H-pyrazol-5-ol)	[Dsim][TFA]	30	93	^65^
5	4,4′-((4-Chlorophenyl)methylene)bis(3-methyl-1-phenyl-1H-pyrazol-5-ol)	ZnFe_2_O_4_@SiO_2_@APTES@DHBS-Cu	60	96	This work
6	Methyl(phenyl)sulfane	MCM‐41‐Adenine‐Zr	55	97	^66^
7	Methyl(phenyl)sulfane	Fe_3_O_4_-AMPD-Pd	20	95	^61^
8	Methyl(phenyl)sulfane	Fe_3_O_4_@chitosan-bound picolinaldehyde Cu	140	93	^67^
9	Methyl(phenyl)sulfane	CoFe_2_O_4_@SiO_2_‐CPTES‐Guanidine‐Cu(II)	20	95	^68^
10	Methyl(phenyl)sulfane	ZnFe_2_O_4_@SiO_2_@APTES@DHBS-Cu	30	97	This work

## Conclusions

In this study, a new type of magnetically recoverable nanocatalyst (ZnFe_2_O_4_@SiO_2_@APTES@DHBS-Cu MNPs) was synthesized. To specify the physicochemical features of the nanocatalyst, various techniques; including, FT-IR, SEM, EDX, XRD, TGA, Map, and VSM analysis were used. This study reported a novel route for the synthesis of an extensive range of bis (pyrazolyl)methanes and sulfoxides with excellent yields. This catalyst can upgrade yields and reaction times in the synthesis of bis (pyrazolyl)methanes and oxidation of sulfides to the sulfoxides compared to many reported methods, with meager leaching amounts of supported catalyst into the reaction mixture. The simple and easy manufacturing method of this catalyst, along with its ability to recover and reuse, makes it economical. The wondrous features of the mentioned organic reactions are high novelty, short reaction times, no use of harmful solvents, simple synthetic procedure, high yields of reactions, facile filtration, and reusability of the catalyst. Furthermore, the synthesized nanocatalyst could be separated easily using an external magnet and reused five times without considerable loss of its activity (Supplementary Information 1).

## Supplementary Information

Supplementary Information.

## Data Availability

All data generated or analysed during this study are included in this published article [and its supplementary information files].

## References

[1] Zhang Y, Pan Z, Wang N, Wang L (2021). Performance of carbon-modified Pd/SBA-15 catalyst for 2-ethylanthraquinone hydrogenation. Mol. Catal..

[2] Noori N, Nikoorazm M, Ghorbani-Choghamarani A (2016). Pd(0)-S-methylisothiourea grafted onto mesoporous MCM-41 and its application as heterogeneous and reusable nanocatalyst for the Suzuki, Stille and Heck cross coupling reactions. J. Porous Mater..

[3] Ghorbani-Choghamarani A, Aghavandi H, Mohammadi M (2021). Mesoporous SBA-15@n-Pr-THAM-ZrO organic–inorganic hybrid: as a highly efficient reusable nanocatalyst for the synthesis of polyhydroquinolines and 2,3-dihydroquinazolin-4 (1h)-ones. J. Porous Mater..

[4] Xu Y (2021). Identification of atomically dispersed Fe-oxo species as new active sites in HZSM-5 for efficient non-oxidative methane dehydroaromatization. J. Catal..

[5] Bestha KK, Abraham JJ, Chelvane JA, Gorige V (2020). Influence of cation distribution on magnetic response of polycrystalline Co_1-__*x*_Ni_*x*_Fe_2_O_4_(0 ≤ *x *≤ 1) ferrites. Phys. Scr..

[6] Tajik S, Shahsavari M, Sheikhshoaie I, Garkani Nejad F, Beitollahi H (2021). Voltammetric detection of sumatriptan in the presence of naproxen using Fe_3_O_4_@ZIF-8 nanoparticles modified screen printed graphite electrode. Sci. Rep..

[7] Kohzadi H, Soleiman-Beigi M (2021). XPS and structural studies of Fe_3_O_4_-PTMS-NAS@Cu as a novel magnetic natural asphalt base network and recoverable nanocatalyst for the synthesis of biaryl compounds. Sci. Rep..

[8] Pourhasan Kisomi R, Shirini F, Golshekan M (2021). Fe_3_O_4_@MCM-41@ZrCl_2_: A novel magnetic mesoporous nanocomposite catalyst including zirconium nanoparticles for the synthesis of 1-(benzothiazolylamino)phenylmethyl-2-naphthols. Appl. Organomet. Chem..

[9] Singh P, Mishra S, Sahoo A, Patra S (2021). A magnetically retrievable mixed-valent Fe_3_O_4_@SiO_2_/Pd0/PdII nanocomposite exhibiting facile tandem Suzuki coupling/transfer hydrogenation reaction. Sci. Rep..

[10] Veisi H (2021). Bio-inspired synthesis of palladium nanoparticles fabricated magnetic Fe_3_O_4_ nanocomposite over Fritillaria imperialis flower extract as an efficient recyclable catalyst for the reduction of nitroarenes. Sci. Rep..

[11] Pormazar SM, Dalvand A (2020). Adsorption of reactive black 5 azo dye from aqueous solution by using amine-functioned Fe_3_O_4_ nanoparticles with L-arginine: Process optimisation using RSM. Int. J. Environ. Anal. Chem..

[12] Muhammad N, Nadeem S (2017). Ferrite nanoparticles Ni–ZnFe_2_O_4_, Mn–ZnFe_2_O_4_ and Fe_2_O_4_ in the flow of ferromagnetic nanofluid. Eur. Phys. J. Plus.

[13] Sheikh, J. R., Gaikwad, V. M., Moon, V. C. & Acharya, S. A. Enhancement in dielectric behavior of (Ni, Zn)Fe_2_O_4_ ferrite. In *AIP Conference Proceedings* vol. 1728 (2016).

[14] Korolkov IV (2021). Boron and gadolinium loaded Fe_3_O_4_ nanocarriers for potential application in neutron cancer therapy. Int. J. Mol. Sci..

[15] Moradi Z, Ghorbani-Choghamarani A (2021). Design and synthesis of Fe_3_O_4_@SiO_2_@KIT-6@DTZ-Pd0 as a new and efficient mesoporous magnetic catalyst in carbon–carbon cross-coupling reactions. Sci. Rep..

[16] Maleki A, Hajizadeh Z, Salehi P (2019). Mesoporous halloysite nanotubes modified by CuFe_2_O_4_ spinel ferrite nanoparticles and study of its application as a novel and efficient heterogeneous catalyst in the synthesis of pyrazolopyridine derivatives. Sci. Rep..

[17] Maleki A, Firouzi-Haji R (2018). L-Proline functionalized magnetic nanoparticles: A novel magnetically reusable nanocatalyst for one-pot synthesis of 2,4,6-triarylpyridines. Sci. Rep..

[18] Hajizadeh Z, Radinekiyan F, Eivazzadeh-keihan R, Maleki A (2020). Development of novel and green NiFe_2_O_4_/geopolymer nanocatalyst based on bentonite for synthesis of imidazole heterocycles by ultrasonic irradiations. Sci. Rep..

[19] Feng X-J (2021). Polydopamine-anchored polyether on Fe_3_O_4_ as magnetic recyclable nanoparticle-demulsifiers. Colloids Surf. A Physicochem. Eng. Asp..

[20] Asri NS (2021). Syntheses of ferrofluids using polyethylene glycol (PEG) coated magnetite (Fe_3_O_4_), citric acid, and water as the working liquid in a cylindrical heat pipe. Nanostruct. Nanoobjects.

[21] Anantharamaiah, P. N., Shashanka, H. M., Kumar, R., Chelvane, J. A. & Sahoo, B. Chemically enabling CoFe_2_O_4_ for magnetostrictive strain sensing applications at lower magnetic fields: Effect of Zn substitution. *Mater. Sci. Eng. B Solid State Mater. Adv. Technol.* **266**, 115080 (2021).

[22] Sahu BN, Sahoo SC, Venkataramani N, Prasad S, Krishnan R (2021). Observation of extraordinarily large magnetization in CoFe_2_O_4_/ZnFe_2_O_4_ bilayers. J. Magn. Magn. Mater..

[23] Eivazzadeh-Keihan R (2021). Novel magnetic organic–inorganic hybrids based on aromatic polyamides and ZnFe_2_O_4_ nanoparticles with biological activity. Sci. Rep..

[24] Lesiak B (2019). Surface study of Fe_3_O_4_ nanoparticles functionalized with biocompatible adsorbed molecules. Front. Chem..

[25] Granath T, Löbmann P, Mandel K (2021). Oxidative precipitation as a versatile method to obtain ferromagnetic Fe_3_O_4_ nano- and mesocrystals adjustable in morphology and magnetic properties. Part. Part. Syst. Charact..

[26] Amiri M, Salavati-Niasari M, Akbari A (2019). Magnetic nanocarriers: Evolution of spinel ferrites for medical applications. Adv. Colloid Interface Sci..

[27] Shi Z, Wang Y, Dong S, Lan T (2021). Comparison of the performance of magnetic targeting drug carriers prepared using two synthesis methods. RSC Adv..

[28] j.msec.2019.110502.pdf.

[29] Maleki A, Niksefat M, Rahimi J, Taheri-Ledari R (2019). Multicomponent synthesis of pyrano[2,3-d]pyrimidine derivatives via a direct one-pot strategy executed by novel designed copperated Fe3O4@polyvinyl alcohol magnetic nanoparticles. Mater. Today Chem..

[30] Maleki A, Varzi Z, Hassanzadeh-Afruzi F (2019). Preparation and characterization of an eco-friendly ZnFe_2_O_4_@alginic acid nanocomposite catalyst and its application in the synthesis of 2-amino-3-cyano-4H-pyran derivatives. Polyhedron.

[31] Maleki A, Hassanzadeh-Afruzi F, Varzi Z, Esmaeili MS (2020). Magnetic dextrin nanobiomaterial: An organic-inorganic hybrid catalyst for the synthesis of biologically active polyhydroquinoline derivatives by asymmetric Hantzsch reaction. Mater. Sci. Eng. C.

[32] Ahghari MR, Soltaninejad V, Maleki A (2020). Synthesis of nickel nanoparticles by a green and convenient method as a magnetic mirror with antibacterial activities. Sci. Rep..

[33] Hajjami M, Sheikhaei S, Gholamian F, Yousofvand Z (2021). Synthesis and characterization of magnetic functionalized Ni and Cu nano catalysts and their application in oxidation, oxidative coupling and various multi-component reactions. Catal. Lett..

[34] Ghorbani-Choghamarani A, Sahraei R, Taherinia Z, Mohammadi M (2020). Cu(I)@Isatin-glycine-boehmite nanoparticles: As novel heterogeneous catalyst for the synthesis and selective oxidation of sulfides. J. Iran. Chem. Soc..

[35] Zhang J, Wei C, Li S, Hu D, Song B (2020). Discovery of novel bis-sulfoxide derivatives bearing acylhydrazone and benzothiazole moieties as potential antibacterial agents. Pestic. Biochem. Physiol..

[36] Pinz MP (2017). 7-Chloro-4-phenylsulfonyl quinoline, a new antinociceptive and anti-inflammatory molecule: Structural improvement of a quinoline derivate with pharmacological activity. Regul. Toxicol. Pharmacol..

[37] He X (2020). Preparation of ceric oxide and cobalt sulfide-ceric oxide/cellulose-chitosan nanocomposites as a novel catalyst for efficient photocatalysis and antimicrobial study. Int. J. Biol. Macromol..

[38] Wang L, Zhang Y, Yao J, Li H (2021). Metal-free synthesis of sulfones and sulfoxides through aldehyde-promoted aerobic oxidation of sulfides. Catal. Lett..

[39] Lu X (2021). EMIMBF4 in ternary liquid mixtures of water, dimethyl sulfoxide and acetonitrile as “tri-solvent-in-salt” electrolytes for high-performance supercapacitors operating at − 70 °C. Energy Storage Mater..

[40] Azizi M, Maleki A, Hakimpoor F, Ghalavand R, Garavand A (2017). A mild, efficient and highly selective oxidation of sulfides to sulfoxides catalyzed by lewis acid–urea–hydrogen peroxide complex at room temperature. Catal. Lett..

[41] Molaei S, Tamoradi T, Ghadermazi M, Ghorbani-Choghamarani A (2019). Ordered mesoporous SBA-15 functionalized with yttrium(III) and cerium(III) complexes: Towards active heterogeneous catalysts for oxidation of sulfides and preparation of 5-substituted 1H-tetrazoles. Appl. Organomet. Chem..

[42] Niakan M, Asadi Z, Masteri-Farahani M (2020). Fe(III)-salen complex supported on dendrimer functionalized magnetite nanoparticles as a highly active and selective catalyst for the green oxidation of sulfides. J. Phys. Chem. Solids.

[43] Al-Adiwish WM (2013). Synthesis, antibacterial activity and cytotoxicity of new fused pyrazolo[1,5-a]pyrimidine and pyrazolo[5,1-c][1,2,4]triazine derivatives from new 5-aminopyrazoles. Eur. J. Med. Chem..

[44] Khanmohammadi-Sarabi F, Ghorbani-Choghamarani A, Aghavandi H, Zolfigol MA (2022). ZnFe_2_O_4_@SiO_2_-ascorbic acid: Green, magnetic, and versatile catalyst for the synthesis of chromeno[2,3-d] pyrimidine-8-amine and quinazoline derivatives. Appl. Organomet. Chem..

[45] Yousofvand Z, Hajjami M, Ghorbani F, Ghafouri-Nejad R (2018). Synthesis of Ni(II)-3,5-dichloro-2-hydroxybenzenesulfonyl chloride supported SBA-15 and its application as a nanoreactor catalyst for the synthesis of diaryl sulfides via reaction of aryl halides with thiourea or S8. J. Porous Mater..

[46] Abbasian AR, Shafiee Afarani M (2019). One-step solution combustion synthesis and characterization of ZnFe_2_O_4_ and ZnFe_1.6_O_4_ nanoparticles. Appl. Phys. A Mater. Sci. Process..

[47] Gholinejad M, Afrasi M, Najera C (2019). Caffeine gold complex supported on magnetic nanoparticles as a green and high turnover frequency catalyst for room temperature A 3 coupling reaction in water. Appl. Organomet. Chem..

[48] Aghajani-Monadi2017_Article_SchiffBaseComplexOfMoSupported.pdf.

[49] Sahoo JK, Rath J, Dash P, Sahoo H (2017). EDTA functionalized magnetic nanoparticle as a multifunctional adsorbent for Congo red dye from contaminated water. AIP Conf. Proc..

[50] Peng H, Wang M, Hu C, Guo J (2020). A new type of MgFe_2_O_4_@cus-aptes nanocarrier for magnetic targeting and light-microwave dual controlled drug release. Int. J. Nanomed..

[51] Villa S, Riani P, Locardi F, Canepa F (2016). Functionalization of Fe3O4 NPs by silanization: Use of amine (APTES) and thiol (MPTMS) silanes and their physical characterization. Materials (Basel).

[52] Thilagavathi, S., Praveen Shanker, N. & Venkateswaran, C. Spin-glass behavior in nanocrystalline ZnFe_2_O_4_ spinel ferrite. In *AIP Conference Proceedings* Vol. 2270 (2020).

[53] Roshani R, Tadjarodi A (2020). Synthesis of ZnFe_2_O_4_ nanoparticles with high specific surface area for high-performance supercapacitor. J. Mater. Sci. Mater. Electron..

[54] Silambarasu A, Manikandan A, Balakrishnan K (2017). Room-temperature superparamagnetism and enhanced photocatalytic activity of magnetically reusable spinel ZnFe_2_O_4_ nanocatalysts. J. Supercond. Nov. Magn..

[55] Filian H, Kohzadian A, Mohammadi M, Ghorbani-Choghamarani A, Karami A (2020). Pd(0)-guanidine@MCM-41: A very effective catalyst for rapid production of bis (pyrazolyl)methanes. Appl. Organomet. Chem..

[56] Kordnezhadian R (2020). Polyethylene glycol-bonded triethylammonium l-prolinate: A new biodegradable amino-acid-based ionic liquid for the one-pot synthesis of bis(pyrazolyl)methanes as DNA binding agents. New J. Chem..

[57] Kohzadian A, Filian H, Kordrostami Z, Zare A, Ghorbani-Choghamarani A (2020). A simple, rapid and effective protocol for synthesis of bis(pyrazolyl)methanes using nickel–guanidine complex immobilized on MCM-41. Res. Chem. Intermed..

[58] Tamoradi T, Ghorbani-Choghamarani A, Ghadermazi M (2019). CoFe_2_O_4_@glycine-M (M= Pr, Tb and Yb): Three green, novel, efficient and magnetically-recoverable nanocatalysts for synthesis of 5-substituted 1H–tetrazoles and oxidation of sulfides in green condition. Solid State Sci..

[59] Molaei S, Ghadermazi M (2021). A green methodology for thioether formation reaction and synthesis of symmetrical disulfides over new heterogeneous Cu attached to bifunctionalized mesoporous MCM-41. Microporous Mesoporous Mater..

[60] Tamoradi T, Ghadermazi M, Ghorbani-Choghamarani AC (2018). Correction to: Synthesis of polyhydroquinoline, 2,3-dihydroquinazolin-4(1H)-one, sulfide and sulfoxide derivatives catalyzed by new copper complex supported on MCM-41. Catal. Lett..

[61] Tamoradi T, Moeini N, Ghadermazi M, Ghorbani-Choghamarani A (2018). Fe_3_O_4_-AMPD-Pd: A novel and efficient magnetic nanocatalyst for synthesis of sulfides and oxidation reactions. Polyhedron.

[62] Niya HF, Hazeri N, Fatahpour M, Roudini P, Shirzaei M (2021). Immobilizing Pd nanoparticles on Fe_3_O_4_@tris (hydroxymethyl) aminomethane MNPs as a novel catalyst for the synthesis of bis (pyrazolyl)methane derivatives. J. Mol. Struct..

[63] Khazaei A, Abbasi F, Moosavi-Zare AR (2014). Tandem cyclocondensation-Knoevenagel-Michael reaction of phenyl hydrazine, acetoacetate derivatives and arylaldehydes. New J. Chem..

[64] Abshirini Z, Zare A (2018). Efficient pseudo five-component synthesis of 4,4′-(arylmethylene)-bis(3-methyl-1-phenyl-1H-pyrazol-5-ol) derivatives promoted by a novel ionic liquid catalyst. Z. Naturforsch. B.

[65] Karami M, Zare A (2018). CHEMISTRY 1,3-disulfonic acid imidazolium trifluoroacetate as a highly efficient and dual-functional catalyst for the pseudo five-component reaction of phenylhydrazine with ethyl acetoacetate and arylaldehydes. Organ. Chem. Res..

[66] Tamoradi T, Ghorbani-Choghamarani A, Ghadermazi M (2018). Synthesis of new zirconium complex supported on MCM-41 and its application as an efficient catalyst for synthesis of sulfides and the oxidation of sulfur containing compounds. Appl. Organomet. Chem..

[67] Fakhri A, Naghipour A (2018). Fe_3_O_4_@chitosan-bound picolinaldehyde Cu complex as the magnetically reusable nanocatalyst for adjustable oxidation of sulfides. Environ. Prog. Sustain. Energy.

[68] Heidari L, Shiri L (2019). CoFe_2_O_4_@SiO_2_-CPTES-Guanidine-Cu(II): A novel and reusable nanocatalyst for the synthesis of 2,3-dihydroquinazolin-4(1H)-ones and polyhydroquinolines and oxidation of sulfides. Appl. Organomet. Chem..

