# Supplementary material for: A new copper-supported zinc ferrite as a heterogeneous magnetic nanocatalyst for the synthesis of bis(pyrazolyl)methanes and oxidation of sulfides
Source: Sci Rep. 2022 Dec 1;12:20775. doi: 10.1038/s41598-022-25170-w (PMC9715624; doi:10.1038/s41598-022-25170-w)
Supplement: Supplementary file 1 — Supplementary Information. [file 41598_2022_25170_MOESM1_ESM.pdf]

Supporting information

A new copper-supported zinc ferrite as a heterogeneous magnetic nanocatalyst for the synthesis of bis(pyrazolyl)methanes and oxidation of sulfides

Arash Ghorbani-Choghamarani,* Hamid Aghavandi, Seyed Mahdi Talebi

Department of Organic Chemistry, Faculty of Chemistry, Bu-Ali Sina University, Hamedan, 6517838683, Iran.

Address correspondence to Arash Ghorbani-Choghamarani, Department of Organic Chemistry, Faculty of Chemistry, Bu-Ali Sina University, Hamedan 6517838683, Iran Tel: +988138282807 Fax: +988138380709 E-mail: a.ghorbani@basu.ac.ir or arashghch58@yahoo.com

Contents		Page
**Figure 1**	**Sulfinyldibenzene:** ^{1}H NMR (250 MHz, $CDCl_3$): 7.22- 7.65 (m, 10H).	**2**
**Figure 2**	**(Methylsulfinyl)benzene:** ^{1}H NMR (250 MHz, $CDCl_3$):2.7 (S, 3H), 7.48-7.93 (m, 5H).	**3**
**Figure 3**	**1-(Butylsulfinyl)butane:** ^{1}H NMR (250 MHz, $CDCl_3$): 0.97(t, 6H), 1.40 (m, 4H), 1.70 (m, 4H), 2.66 (t, 4H).	**4**
**Figure 4**	**4,4'-((4-Tolyl)methylene)bis(3-methyl-1-phenyl-1H-pyrazol-5-ol):** ^{1}H NMR (250 MHz, $CDCl_3$): 2.29 (s, 6H), 2.41 (s, 3H), 4.79 (s, 1H), 7.09-7.69 (m, 14H), 7.72 (s, 2H, OH) ppm.	**5**
**Figure 5**	**4,4'-((4-Methoxyphenyl)methylene)bis(3-methyl-1-phenyl-1H-pyrazol-5-ol):** ^{1}H NMR (250 MHz, $CDCl_3$): δ 2.25 (s, 6H), 3.75 (s, 3H), 4.77 (s, 1H), 6.77-7.95 (m, 14H, Ar), 9.87 (b, 2H, OH).	**6**

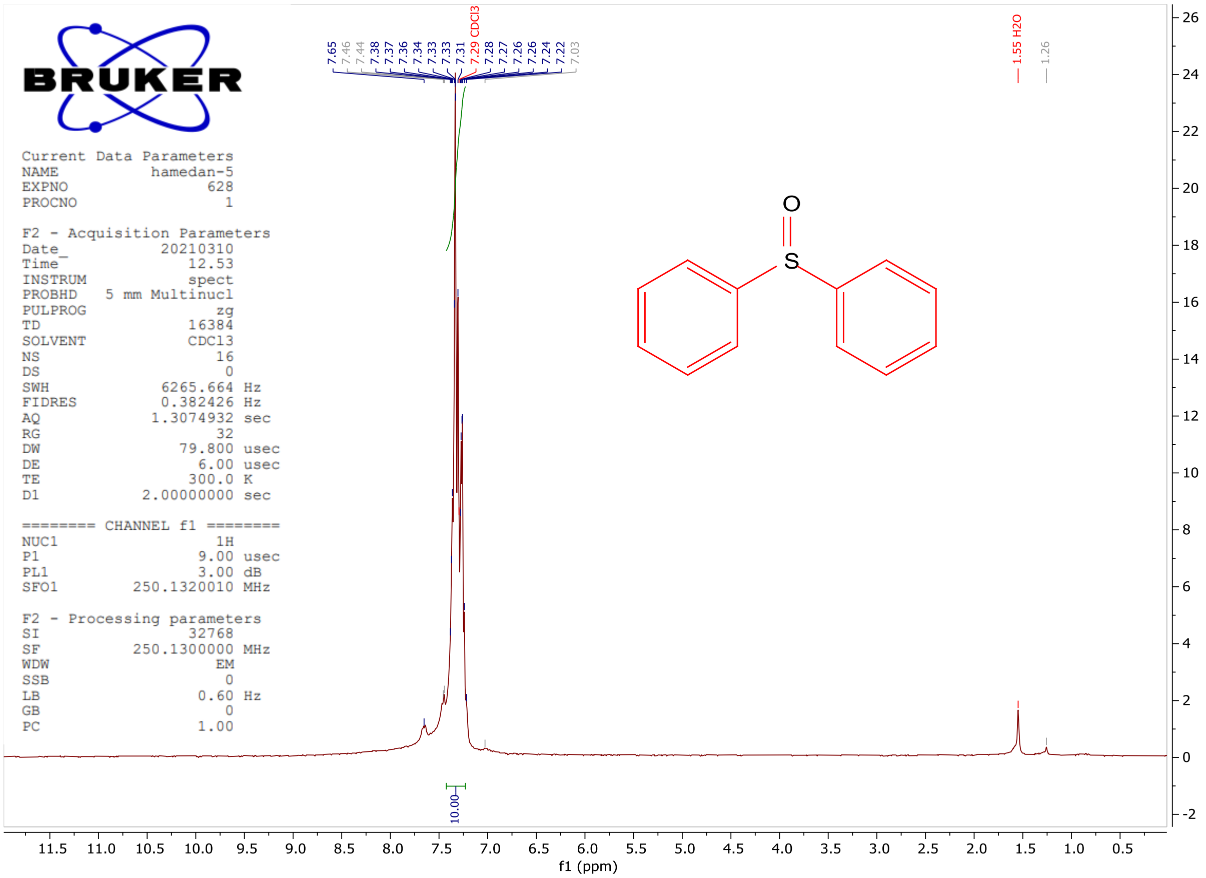

Figure 1: ^{1}H-NMR Sulfinyldibenzene (Table 4, Entries 9).

Sulfinyldibenzene: ^{1}H NMR (250 MHz, $CDCl_3$): 7.22- 7.65 (m, 10H).

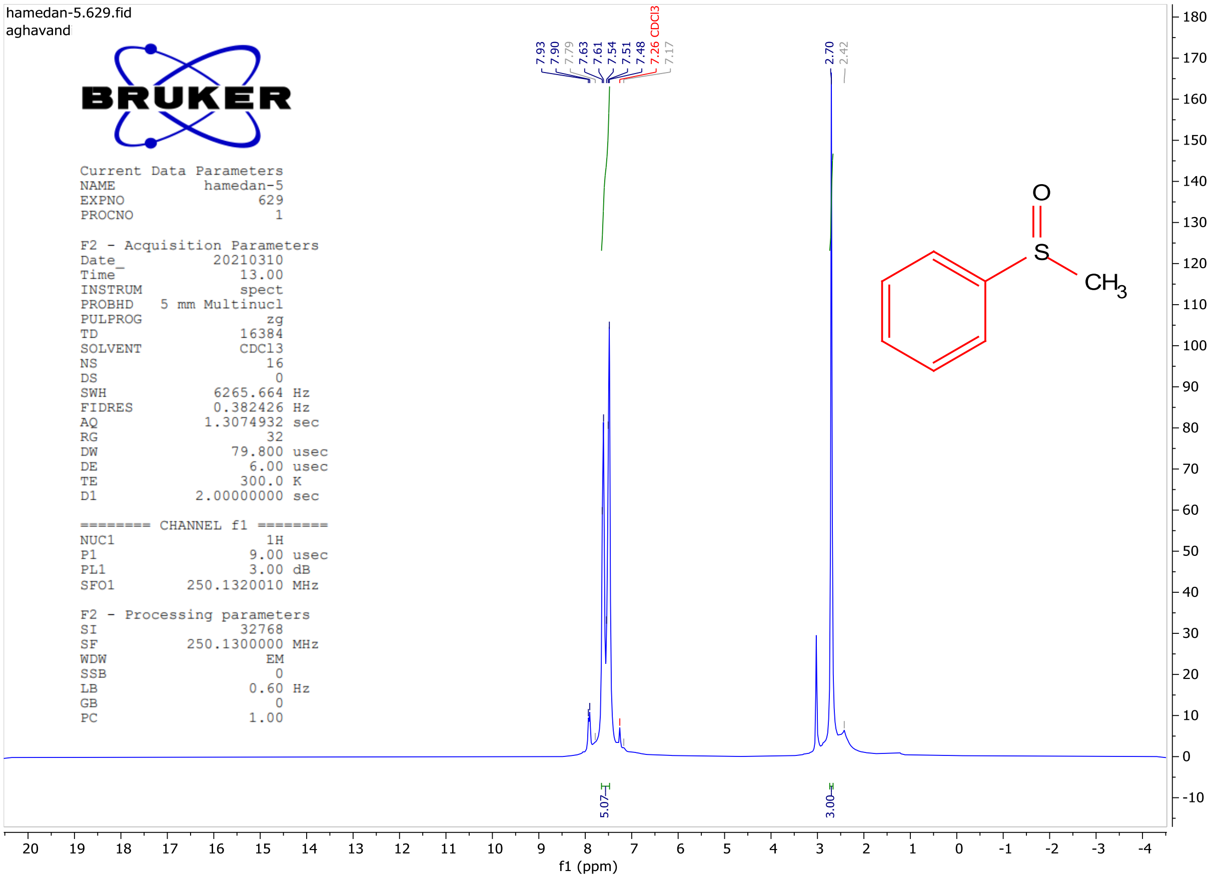

Figure 2: ^{1}H-NMR (methylsulfinyl)benzene (Table 4, Entries 1).

(Methylsulfinyl)benzene: ^{1}H NMR (250 MHz, $CDCl_3$):2.7 (S, 3H), 7.48- 7.93 (m, 5H).

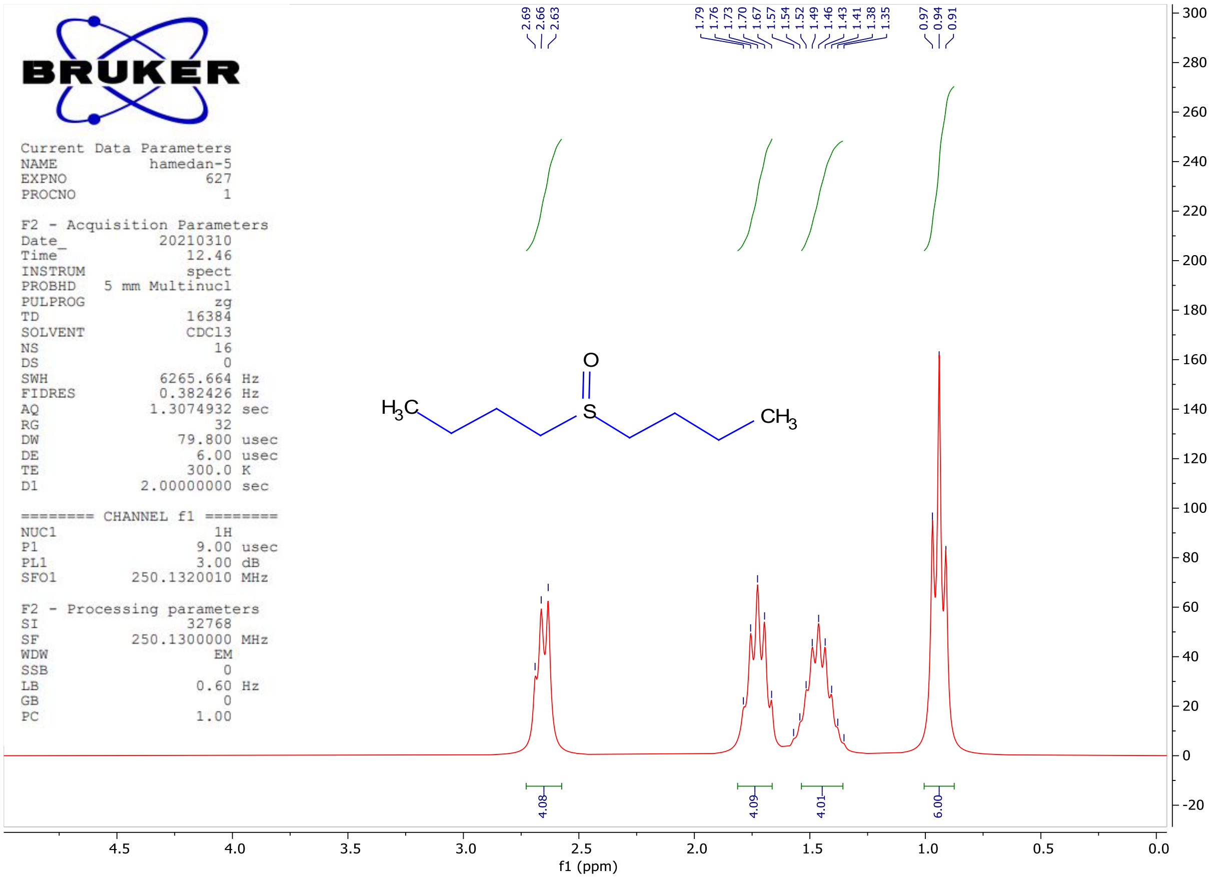

Figure 3: ^{1}H-NMR 1-(butylsulfinyl)butane (Table 4, Entries 6).

1-(Butylsulfinyl)butane: ^{1}H NMR (250 MHz, $CDCl_3$): 0.94(t, 7.5 Hz, 6H), 1.40 (m, 4H), 1.70 (m, 4H), 2.66 (t, 4H).

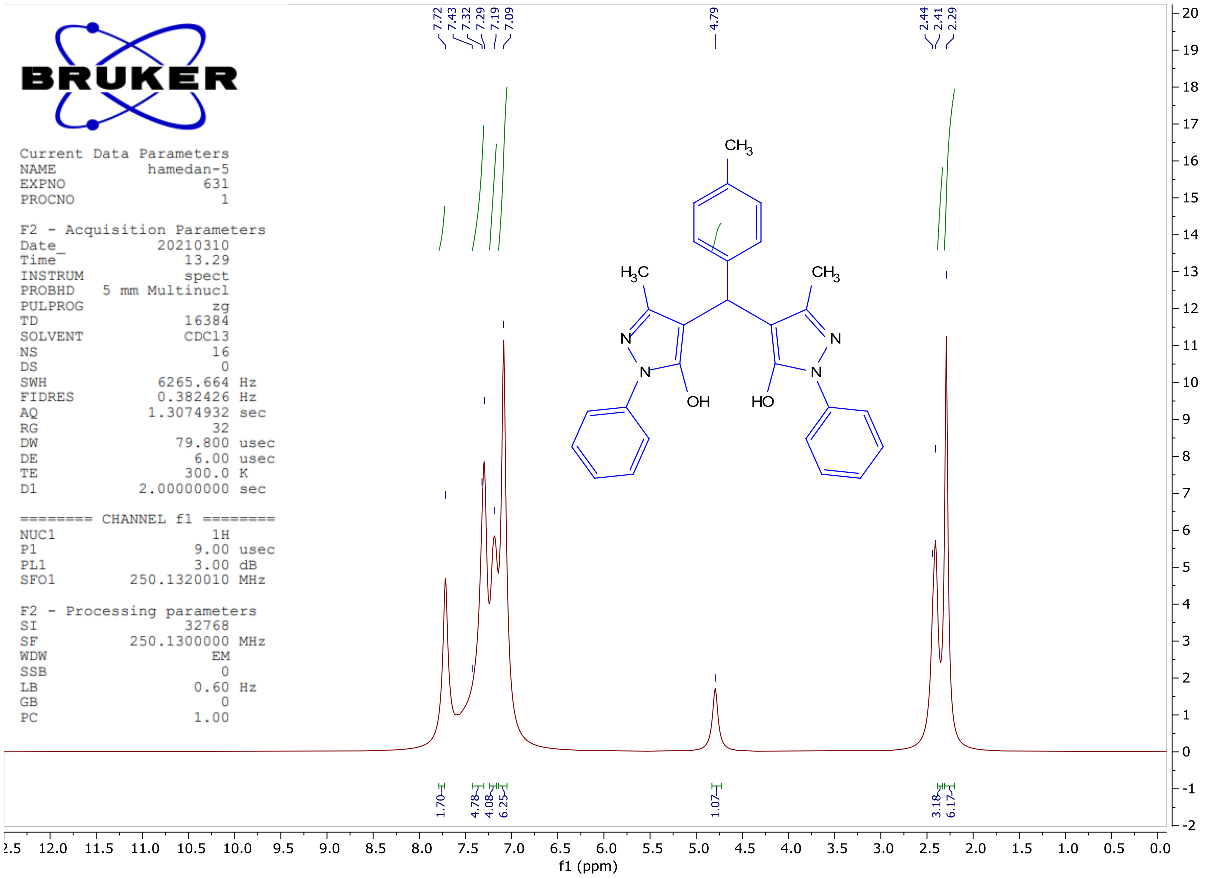

Figure 4: ^{1}H-NMR 4,4'-(p-tolylmethylene)bis(3-methyl-1-phenyl-1H-pyrazol-5-ol) (Table 2, Entries 6).

4,4'-((4-tolyl)methylene)bis(3-methyl-1-phenyl-1H-pyrazol-5-ol): ^{1}H NMR (250 MHz, $CDCl_3$): 2.29 (s, 6H), 2.41 (s, 3H), 4.79 (s, 1H), 7.09-7.69 (m, 14H), 7.72 (s, 2H) ppm.

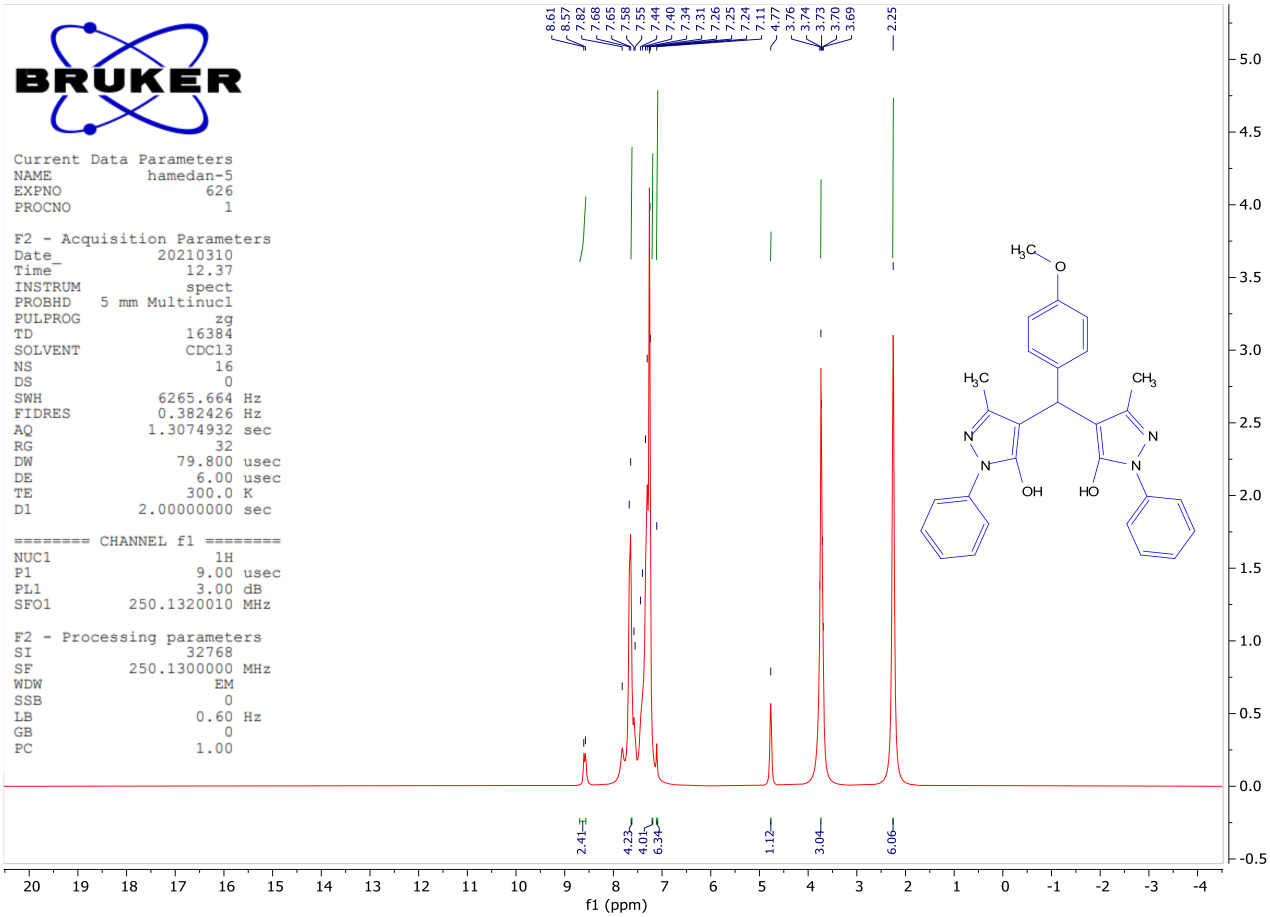

Figure 5: ^{1}H-NMR 4,4'-((4-methoxyphenyl)methylene)bis(3-methyl-1-phenyl-1H-pyrazol-5-ol) (Table 2, Entries 7).

4,4'-((4-methoxyphenyl)methylene)bis(3-methyl-1-phenyl-1H-pyrazol-5-ol): ^{1}H NMR (250 MHz, $CDCl_3$): δ 2.25 (s, 6H), 3.75 (s, 3H), 4.77 (s, 1H), 6.77-7.95 (m, 14H), 8.57-8.61 (b, 2H).
